# Complete Genome Sequence of the *Escherichia coli* Phage Ayreon

**DOI:** 10.1128/genomeA.01354-17

**Published:** 2018-01-11

**Authors:** Marnix Vlot, Franklin L. Nobrega, Che F. A. Wong, Yue Liu, Stan J. J. Brouns

**Affiliations:** a Laboratory of Microbiology, Department of Agrotechnology and Food Sciences, Wageningen University, Wageningen, The Netherlands; b Department of Bionanoscience, Kavli Institute of Nanoscience, Delft University of Technology, Delft, The Netherlands

## Abstract

We report the whole-genome sequence of a new *Escherichia coli* temperate phage, Ayreon, comprising a linear double-stranded DNA (dsDNA) genome of 44,708 bp.

## GENOME ANNOUNCEMENT

Phage Ayreon was isolated from pond water samples in Wageningen, The Netherlands (51°58′9.2″N, 5°40′43.1″E) using *Escherichia coli* strain KD471 as the host, a derivative of *E. coli* K-12 derivative strain KD263 (1) lacking clustered regularly interspaced short palindromic repeat (CRISPR)-associated genes *Cas1* and *Cas2*. Infected cultures were given time to allow for lysogeny. Formed lysogens were induced by using both UV exposure and mitomycin C, demonstrating that phage Ayreon is a temperate phage. Transmission electron microscopy revealed icosahedral capsids (±57 nm) and long flexible tails (±120 nm), which are characteristic of *Siphoviridae* (2).

Phage DNA was extracted using the SDS-proteinase K protocol previously described (3). Library preparation and sequencing were performed by BaseClear (The Netherlands) using the Illumina HiSeq 2500 platform. About 1,282,954 short reads were generated with a mean 3,478-fold coverage of the genome. The resulting sequences were *de novo* assembled using the CLC Genomics Workbench version 8.5.1. The contig sequences were corrected with Pilon version 1.11 (4) and linked using the SSPACE Premium scaffolder version 2.3 (5). Gapped regions within the scaffolds were partially closed in an automated manner using GapFiller version 1.10 (6). Annotation and identification of open reading frames (ORFs) were performed using the Rapid Annotations using Subsystems Technology (RAST) annotation server (7) followed by manual curation of all predicted proteins against the NCBI protein database using BLASTp (8) and Pfam domain searches (9). tRNAs were predicted with tRNAscan-SE version 1.21 (10), and promoters and terminators were identified using motif searches for TTGACAN(15,18)TATAAT with a maximum of two mismatches and ARNold (11), respectively. The genome packaging strategy was predicted by phylogenetic analysis of the large terminase subunit (12).

Phage Ayreon has a linear double-stranded DNA with a genome size of 44,708 bp and a G+C content of 50.1%, which is very similar to the G+C content of its bacterial host (50.8%). The phage has 59 predicted ORFs, of which 25 could not be assigned to a function. Three predicted promoters and 13 predicted Rho-independent terminators were identified; no tRNAs were identified. Phylogenetic analysis of the large terminase subunit suggested that phage Ayreon uses cohesive end site (*cos*) packaging; *cos* sites are expected to be located within ~1 kbp upstream of the small terminase subunit (13). Considering this, the genome of phage Ayreon was opened so that it would begin with the small terminase subunit and end with the expected location of the *cos* site.

The highest degree of similarity was observed with phage cdtI (GenBank accession number AB285204), a cyclomodulin producing prophage (14), with 78% coverage and 96% identity. Phage cdtI contains a gene cluster associated with virulence, encoding the CdtA, CdtB, and CdtC subunits of the cdtI holotoxin. Whole-genome alignment of phage Ayreon and cdtI prophage demonstrates that the *cdtI* gene cluster is absent from phage Ayreon. Global alignment with the attP integration site of phage cdtI shows pairwise identity of >92%, indicating that phage Ayreon integrates into the gene coding for peptide chain release factor RF-3 in the host genome (14).

### Accession number(s).

The complete genome sequence of Ayreon has been deposited in GenBank under the accession no. MF807953.
